# Heparanase 2 expression inversely correlates with bladder carcinoma grade and stage

**DOI:** 10.18632/oncotarget.8003

**Published:** 2016-03-09

**Authors:** Miriam Gross-Cohen, Sari Feld, Inna Naroditsky, Ofer Nativ, Neta Ilan, Israel Vlodavsky

**Affiliations:** ^1^ Cancer and Vascular Biology Research Center, Rappaport Faculty of Medicine, Technion, Haifa, Israel; ^2^ Department of Pathology, Rambam Health Care Campus, Haifa, Israel; ^3^ Department of Urology, Bnai-Zion Medical Center, Haifa, Israel

**Keywords:** Heparanase 2, bladder cancer, LOX, tumor growth, tumor grade

## Abstract

While the pro-tumorigenic function of heparanase is well taken, the role of its close homolog, heparanase 2 (Hpa2) in cancer is by far less investigated. Utilizing immunohistochemical analysis we found that Hpa2 is expressed by normal bladder transitional epithelium and its levels are decreased substantially in bladder cancer. Notably, tumors that retain high levels of Hpa2 were diagnosed as low grade (p=0.001) and low stage (p=0.002), suggesting that Hpa2 is required to preserve cell differentiation and halt cell motility. Indeed, migration of 5637 bladder carcinoma cells was attenuated significantly by exogenous addition of purified Hpa2, and over expression of Hpa2 in 5637 cells resulted in smaller tumors that were diagnosed as low grade. We also noted that tumors produced by Hpa2 over expressing cells are abundantly decorated with stromal cells and collagen deposition evident by Masson's/Trichrome staining, correlating with a marked increase in lysyl oxidase (LOX) staining. The association between Hpa2 and LOX was further confirmed clinically, because of the 16 cases that exhibited strong staining of Hpa2, 14 (87.5%) were also stained strongly for LOX (p=0.05). Collectively, our results suggest that Hpa2 functions as a tumor suppressor in bladder cancer, maintaining cellular differentiation and decreasing cell motility in a manner that appears to be independent of regulating heparanase activity.

## INTRODUCTION

Compelling evidence tie heparanase levels with tumor initiation, growth, metastasis, and chemo resistance [1–7], making it an attractive target for the development of anti-cancer drugs [8–10]. Heparanase 2 (Hpa2) was identified as a close homolog of heparanase based on sequence similarity [11], but its function in tumorigenesis is by far less investigated. Like heparanase, Hpa2 binds heparin/HS with high affinity but lacks HS-degrading activity [12], the hall mark of heparanase. In fact, Hpa2 exhibits even higher affinity to HS compared with heparanase, suggesting that Hpa2 may inhibit heparanase activity by competition for the HS substrate [12]. Previously, we have reported that Hpa2 expression is markedly elevated in head and neck carcinoma compared with the normal epithelium, correlating with prolonged time to disease recurrence (follow-up to failure) and inversely correlating with tumor cell dissemination to regional lymph nodes [12], suggesting that Hpa2 functions as a tumor suppressor. Here, we examined Hpa2 expression in bladder carcinoma and correlated Hpa2 levels with tumor grade and stage. We found that Hpa2 is expressed at high levels in the normal bladder transitional epithelium whereas its expression is markedly decreased in bladder carcinoma. Notably, tumors that retain high levels of Hpa2 exhibit higher degree of cell differentiation (low grade) and are less invasive (low stage). Over expression of Hpa2 in 5637 human bladder carcinoma cells resulted in tumors smaller in size that recapitulate the clinical manifestation and are characterized as low grade vs. high grade of control tumors. These results suggest that Hpa2 functions as a tumor suppressor in bladder cancer.

## MATERIALS AND METHODS

### Patients

Bladder cancer tissue array of 75 human tumors (in duplicates; 150 samples per array) having grade, stage and TNM characterization was purchased from Biomax.US (BL1501; Rockville, MD). Of these, 69 were diagnosed with bladder cancer (transitional cell carcinoma, 50; adenocarcinoma, 12; squamous cell carcinoma, 7), 5 exhibited chronic cystitis, and 1 had low grade leiomyosarcoma (Table 1). The latter 6 biopsies were discarded from further analysis. The study also included 23 patients with bladder cancer that were diagnosed in the Department of Urology, Bnai-Zion Medical Center, Haifa, Israel, whose archival paraffin-embedded pathological material was available for immunohistochmical analysis. The study protocol was approved by the Bnai-Zion Medical Center Helsinki Committee Institutional Review Board (IRB). Being a retrospective study that includes data retrieved from medical records and paraffin blocks, the local IRB do not require individual patients approval by signing a written inform consent. These patients underwent surgical removal of the tumors [i.e. transurethral resection of bladder wall lesions (TUR-T)] that were detected mostly following evaluation of painless hematuria but also due to irritated symptoms or as a results of incidental radiological finding. In cases of lamina propria invasion (stage T1) and /or high grade lesion, re-TUR-T was performed to exclude muscle invasive disease. Histologic sections of the resected tumors were stained with hematoxylin and eosin and were analyzed by uro-pathologist to determine histologic grade and tumor stage.

**Table 1:** Clinical description of patients

Parameter	Number of patients (out of 69)	%
**Gender:**		
Female	16	23
Male	53	77
**Age:**		
Min-31		
Max-85		
Median-62		
**Type of tumor:**		
Transitional Cell Car.	50	72
Adenocarcinoma	12	17
Squamous Cell Car.	7	11
*Grade:		
1	19	29
2	21	31
3	27	40
**Stage:**		
I	13	19
II	40	58
III	16	23
**Hpa2 staining intensity:		
0	4	6
1	26	38
2	22	32
3	16	24
**LOX staining intensity:		
0	3	4
1	16	24
2	26	38
3	23	34

* Data on two patients was missing;

** Data on one patient was missing

### Cells and cell culture, immunoblotting, and heparanase activity assay

5637 bladder carcinoma cells were grown in Dulbecco's modified Eagle's medium (Biological Industries, Beit Haemek, Israel) supplemented with 10% fetal bovine serum and antibiotics. Cells were passed in culture no more than 2 months after being thawed from authentic stocks (ATCC). Cells were infected with control empty vector (Vo) or Hpa2 gene construct, selected with Puromycin (2 μg/ml; Invitrogen), expended and pooled. Cell clones were isolated by limiting dilution and clones expressing high levels of Hpa2 were evaluated by immunoblotting, carried out essentially as described [12]. Cell clones of control (Vo) 5637 cells were selected randomly. Preparation of dishes coated with sulfate labeled extracellular matrix (ECM) and determination of heparanase enzymatic activity (i.e., release of sulfate labeled heparan sulfate degradation fragments) were carried out essentially as described previously [12].

### Antibodies and reagents

Anti-Hpa2 polyclonal (Ab 58) and monoclonal (20c5) antibodies have been described previously [12]. Immunohistochemical-grade anti-LOX polyclonal antibody was kindly provided by Dr. Peleg Hasson (Technion, Israel) [13]. Anti-actin monoclonal antibody and Masson's/Trichrome staining kit were purchased from Sigma. Anti-E-cadherin and anti-β-catenin antibodies were purchased from Santa Cruz Biotechnology (Santa Cruz, CA).

### Tumorigenicity and immunohistochemistry

Control (Vo; clone #12) and Hpa2 over expressing cells (Hpa2; Clone #B4) were detached with trypsin/EDTA, washed with PBS, and brought to a concentration of 5×10^7^ cells/ml in 50% Matrigel. Cell suspension (5×10^6^/0.1 ml) was inoculated subcutaneously at the right flank of 6 week-old NOD/SCID mice (n=7). Xenografts size was determined by externally measuring tumors in 2 dimensions using a caliper. At the end of the experiment, mice were sacrificed; tumor xenografts were removed, weighed, and fixed in formalin. Paraffin-embedded 5 μm sections were subjected to immunostaining with the indicated antibody using the Envision kit according to the manufacturer's (Dako) instructions, as described previously [12]. Immunostained specimens were examined by a senior pathologist (IN) who was blind to clinical data of the patients and was scored according to the intensity of staining (0: none, +1: weak; +2: moderate; +3: strong). Staining applying the above procedure but lacking the primary antibody yielded no detectable staining.

### Statistics

Data are presented as means ± SE. Statistical significance was analyzed by Sommer's D test or 2-tailed Student's *t* test. Values of *P* ≤ 0.05 were considered significant. Data sets passed D'Agostino-Pearson normality (GraphPad Prism 5 utility software). All experiments were repeated at least 3 times with similar results.

## RESULTS

### Hpa2 levels are decreased in bladder carcinoma

**Figure 1:**
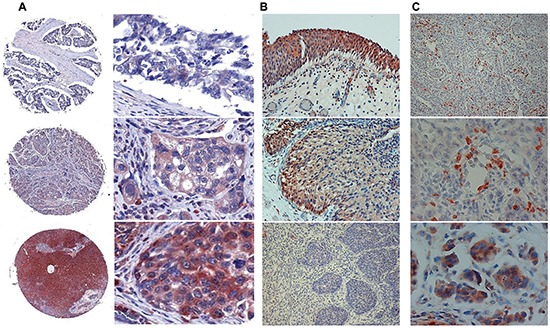
Hpa2 levels are decreased in bladder cancer **A.** Bladder tissue array. Tissue array containing 69 biopsies of human bladder tumors was subjected to immunostaining applying anti-Hpa2 polyclonal antibody. Shown are representative photomicrographs of tumors that exhibit no or very weak staining (0-1; upper panels), moderate (+2, middle panels) or strong (+3; lower panels) staining. Original magnifications: left panels: x5, right panels: x40. **B–C.** Bladder biopsies. Bladder tumor biopsies were subjected to immunostaining applying anti-Hpa2 polyclonal antibody. Strong Hpa2 staining is detected in normal transitional epithelium of the bladder (B, upper panel) which is decreased substantially in bladder carcinomas (B, middle and lower panels). Original magnifications: x10. Hpa2 staining is also detected in immune cells within tumors (C, upper and middle panels), including macrophages giant cells (C, lower panel). Original magnifications: upper panel: x10, middle and lower panels: x40.

**Figure 2:**
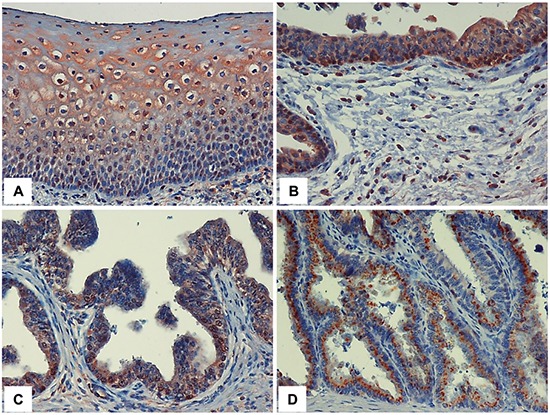
Hpa2 staining in the epithelium of normal tissues adjacent to the bladder tumor Bladder tumor biopsies were subjected to immunostaining applying anti-Hpa2 polyclonal antibody and specimens were examined for the presence of tissues adjacent to the bladder tumor. Strong Hpa2 staining is evident in normal squamous epithelium of the bladder (metaplasia; **A.**) urethra **B.** prostate **C.** and seminal vessels **D.** epithelium. Original magnifications: x40.

In order to reveal the expression and significance of Hpa2 in bladder cancer we subjected a bladder cancer tissue array to immunostaining applying anti-Hpa2 antibody. The staining showed distinct staining intensities among patients, exhibiting none or very weak (0-1; Figure 1A, upper panels), moderate (+2; Figure 1A, middle panels) or strong (+3; Figure 1A, lower panels) staining. In order to confirm this staining pattern we utilized human bladder carcinoma biopsies collected at the Bnai-Zion Medical Center, that, unlike the array samples, contain large area of the tumor and in some cases also adjacent normal tissue. Notably, we found that Hpa2 is being expressed at high levels by the normal bladder transitional epithelium (Figure 1B, upper panel), which is decreased substantially in the bladder carcinoma (Figure 1B, middle and lower panels). In some cases, we observed a very weak staining of Hpa2 in the carcinoma cells but strong staining in seemingly inflammatory cells (Figure 1C, upper and middle panels), including macrophages giant cells (Figure 1C, lower panel). Occasionally, in an attempt to remove the entire tumor mass, the biopsies also included adjacent normal tissues other than the bladder. We could thus detect strong Hpa2 staining not only in the normal bladder transitional epithelium (Figure 1B) but also in squamous epithelium of the bladder (Figure 2A), transitional epithelium of the urethra (Figure 2B), and epithelium of the prostate (Figure 2C) and seminal vessels (Figure 2D). This suggests that unlike head and neck [12], Hpa2 is being expressed at apparently high levels by the normal bladder epithelium as well as normal epithelium of other organs, and its levels are decreased substantially or absent in bladder carcinoma, an expression pattern typical of a tumor suppressor.

### High levels of Hpa2 are associated with low grade and low stage tumors

In order to reveal the significance of Hpa2 in bladder carcinoma we examined the association between Hpa2 levels and tumor grade (i.e., cell differentiation) and stage (i.e., tumor invasiveness). Importantly, tumors that retained high levels of Hpa2 (+3) exhibited higher degree of cell differentiation and were low-grade (Table 2). Thus, of the 16 patients that were stained strongly for Hpa2 (+3), 15 (94%) were diagnosed as grade 1 or 2 and only 1 (6%) was diagnosed as grade 3, differences that are statistically highly significant (p<0.001). The inverse correlation between Hpa2 staining intensity and tumor grade was also obtained in the more homogenous group of patients diagnosed with transitional cell carcinoma (Table 3; p<0.001). Moreover, tumors that retained high levels of Hpa2 immunoreactivity were diagnosed as low stage (Table 4). Here, 77% (10/13) of the Stage I patients stained strongly for Hpa2 (2+3), whereas the majority (12/16; 75%) of stage III tumors exhibited no (0) or weak (+1) staining of Hpa2, differences that are statistically highly significant (p<0.002). The inverse correlation between Hpa2 staining intensity and tumor stage was similarly observed in the more homogenous group of patients diagnosed with transitional cell carcinoma (Table 5; p<0.001).

**Table 2:** Hpa2 staining intensity inversely associates with bladder tumor grade

Grade	Hpa2 intensity (%)		Total
	0+1+2	3.00	
1+2	25 (50)	15 (94)	40
3.00	25 (50)	1 (6)	26
Total	50	16	66

Somers'd R = −0.38, P<0.001.

**Table 3:** Hpa2 staining intensity inversely associates with transitional cell carcinoma grade

Grade	Hpa2 intensity (%)		Total
	0+1+2	3.00	
1+2	17 (51)	15 (100)	32
3.00	16 (49)	0 (0)	16
Total	33	15	48

Somers'd R = −0.48, P<0.001.

**Table 4:** Hpa2 staining intensity inversely associates with bladder tumor stage

Stage	Hpa2 intensity		Total
	0+1	2+3	
I	3 (23)	10 (77)	13
II	15 (38)	24 (62)	39
III	12 (75)	4 (25)	16
Total	30	38	68

Somers'd R = −0.33, p<0.002.

**Table 5:** Hpa2 staining intensity inversely associates with transitional cell carcinoma stage

Stage	Hpa2 intensity		Total
	0+1	2+3	
I	5 (23)	8 (77)	13
II	24 (38)	7 (62)	31
III	5 (75)	0 (25)	5
Total	34	15	49

Somers'd R = −0.37, p<0.001.

### Over expression of Hpa2 in 5637 bladder carcinoma cells results in low grade tumor xenografts

**Figure 3:**
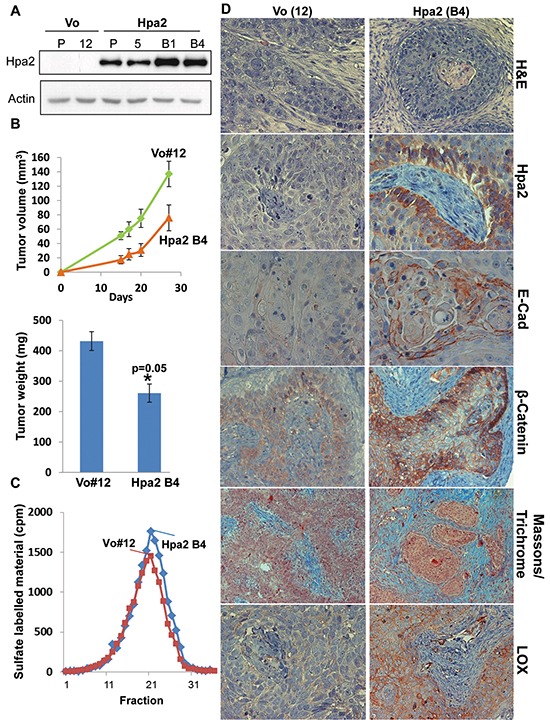
Over expression of Hpa2 in 5637 bladder carcinoma cells results in low grade tumors and attenuates tumor growth **A.** Immunoblotting. 5637 cells were infected with control (Vo) and Hpa2 gene constructs, selected with Puromycin (2 μg/ml; Invitrogen), expended and pooled (P). Cell clones were isolated by limiting dilution and clones exhibiting higher Hpa2 expression (B4) vs. the pool of cells evident by immunoblotting were used for subsequent experiments. Clones of control (Vo) cells were selected randomly (i.e., Vo #12). **B.** Tumor xenografts. Control (Vo #12) and Hpa2-B4 5637 cells (5×106) were implanted subcutaneously in NOD/SCID mice and tumor volume was inspected (upper panel). At termination, tumor xenografts were collected, weighed (lower panel) and formalin-fixed for histological and immunohistochemical analyses. **C.** Heparanase activity. Control (Vo #12) and Hpa2-B4 cells (2×106) were subjected to three freeze/thaw cycles and the resulting cell extracts were applied onto dishes coated with sulfate-labeled ECM and incubated for 6 hours. The incubation medium was then collected and evaluated for heparanase activity as described in ‘Materials and Methods’. **D.** Paraffin-embedded 5 micron sections were subjected to histological and immunohistochemical examination. Shown are representative images of hematoxylin & eosin (H&E) staining (upper panels), and immunostaining applying antibodies directed against Hpa2 (second panels), E-cadherin (third panels), β-catenin (fourth panels), and LOX (lower panels). Original magnifications: x40. Masson's/Trichrome staining is shown in the fifth panels (original magnification x20).

**Figure 4:**
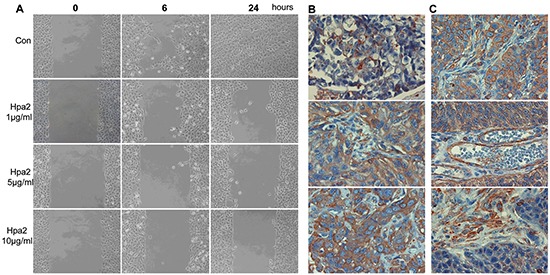
**A.** Hpa2 attenuates 5637 cell migration. Parental 5637 bladder carcinoma cells were plated in ibidi cell migration inserts apparatus (Planegg, Germany) until confluent. The barrier was then removed, cell cultures were washed and changed to serum-free medium, and migration into the defined cell-free gap was inspected in the absence (Con) or presence of the indicated concentration of purified Hpa2. Shown are representative photomicrographs taken before (0), 6, and 24 hours after the addition of Hpa2. Note that cell migration and wound closure is attenuated prominently by exogenous Hpa2. **B.** LOX staining. The bladder tissue array was subjected to immunostaining applying anti-LOX polyclonal antibody. Shown are representative photomicrographs of tumors that show weak (+1; upper panel), moderate (+2, middle panel) or strong (+3; lower panel) staining. **C.** LOX staining is also found enriched at apparently cell-cell borders (upper panel) and in endothelial (middle panel) and stromal cells (lower panel) within tumors. Original magnifications: x40.

In order to substantiate the association between high Hpa2 levels and low grade/stage tumors we over expressed Hpa2 in 5637 human bladder carcinoma cells and cell clones expressing high levels of Hpa2 were selected on the basis of immunoblotting (Figure 3A). Tumor xenografts produced by 5637 cells over expressing Hpa2 were significantly smaller (Figure 3B). Heparanase enzymatic activity was practically identical in control (Vo #12) and Hpa2 (#B4) cells (Figure 3C), suggesting that the decrease in tumor growth is not due to inhibition of heparanase activity by Hpa2. Histological examination revealed that tumors produced by Hpa2 over expressing cells are low grade vs. high grade of tumors produced by control (Vo) cells (Figure 3D, upper panels), also associating with abundant epithelial cadherin staining at cell-cell junctions (E-Cad; Figure 3D, third panel). Likewise, localization of β-catenin at cell-cell contacts was increased substantially in cells over expressing Hpa2 (Figure 3D, fourth panels). We also noted that Hpa2 over expression is associated with higher abundance of stromal cells and collagen deposition evident by Masson's/Trichrome staining (Figure 3D, fifth panels, blue), and suspected that the increase in collagen deposition may involve lysyl oxidase (LOX), an enzyme that is strongly implicated in collagen deposition and tissue fibrosis [14, 15]. Indeed, immunostaining (Figure 3D, lower panels) revealed a strong induction of LOX expression in tumor xenografts produced by cells over expressing Hpa2. We further examined the motility of 5637 cells in a wound scratch assay and found that wound closure was attenuated significantly following the addition of purified Hpa2 vs. untreated cells (Figure 4A).

### Association between Hpa2 and LOX expression in bladder cancer

In order to further reveal the clinical significance of LOX in bladder cancer we next subjected the bladder tissue array to immunostaining applying anti-LOX antibody. We found that most biopsies are stained positive for LOX, exhibiting weak (Figure 4B, upper panel), moderate (Figure 4B, middle panel) or strong (Figure 4B, lower panel) staining. In some cases, LOX staining was most evident at areas of cell-cell junctions rather than diffused in the cytoplasm (Figure 4C, upper panel) and was also abundant in endothelial (Figure 4C, second panel) and stromal cells (Figure 4C, lower panel). Most importantly, LOX staining correlated with Hpa2 staining (Table 6). Hence, of the 16 cases that exhibited strong staining of Hpa2, 14 (87.5%) were also stained strongly for LOX (p=0.05).

**Table 6:** Hpa2 staining intensity correlates with LOX staining intensity

Hpa2	LOX		Total
	0+1	2+3	
0+1+2	17 (33)	35 (67)	52
3.00	2 (12.5)	14 (87.5)	16
Total	19	49	68

p=0.05.

Collectively, our results suggest that Hpa2 functions as a tumor suppressor in bladder cancer, maintaining cellular differentiation and decreasing cell motility. Furthermore, we show for the first time that Hpa2 is involved in gene expression (i.e., LOX, E-cadherin) in apparently heparanase-independent manner.

## DISCUSSION

Bladder cancer is ranked fifth among cancers in men in Western countries and is the most common cancer of the urinary tract, with estimated 380,000 new cases and ~150,000 deaths per year worldwide [16]. Non-muscle-invading bladder cancers frequently recur, but infrequently progress to invasion (10-15%), and five-year survival reaches 90% [16]. In contrast, the five-year survival of muscle-invading (stage T2 and above) bladder cancer is less than 50%, and treatment has not advanced for several decades [17]. Improved treatment requires detailed understanding of the pathogenesis and molecular biology of bladder cancer. Here we show that Hpa2 is expressed at high levels by the normal human bladder epithelium (Figure 1B). This is in agreement with high levels of Hpa2 mRNA expression in the mouse bladder [18, 19]. Notably, Hpa2 expression was prominently reduced in bladder carcinomas (Figure 1), an expression pattern typical of a tumor suppressor protein. Moreover, tumors that retained high levels of Hpa2 exhibited higher degree of cell differentiation (low grade; Tables 2, 3) and were less invasive (low stage; Tables 4, 5), further implying that Hpa2 functions to maintain a normal phenotype. This notion is supported by the 5637 bladder carcinoma cell model, *in vitro* and *in vivo*. Utilizing a cell scratch assay we found that migration and wound closure was attenuated markedly by the addition of purified Hpa2 to 5637 cells (Figure 4A), suggesting that Hpa2 affects cell motility. Furthermore, over expression of Hpa2 in 5637 cells resulted in smaller tumor xenografts that were diagnosed as low grade tumors (Figure 3B, 3D), thus recapitulating the clinical association between Hpa2 levels and cell differentiation. Hpa2 is also detected in inflammatory cells within tumors (Figure 1C) but revealing its function in inflammation requires in depth investigations.

Previously we have reported that Hpa2 inhibits the enzymatic activity of heparanase when purified active heparanase and Hpa2 proteins were combined together [12]. Notably, however, heparanase activity appeared unchanged in 5637 cells over expressing Hpa2 (Figure 3C), suggesting that Hpa2 can function in a manner other than heparanase regulation. Indeed, we found that Hpa2 is involved in the regulation of gene expression, best exemplified by enhanced LOX and E-cadherin expression in 5637 bladder carcinoma cells over expressing Hpa2 (Figure 3D). The association between Hpa2 and LOX levels was further confirmed clinically, because the majority of bladder carcinomas that exhibited strong staining of Hpa2 also showed strong staining of LOX (Table 6).

The role of LOX in bladder cancer has not been so far resolved. However, over expression of the LOX-related enzymes, LOXL1 and LOXL4, in 5637 cells antagonized Ras activation and reduced Erk phosphorylation, suggesting that these enzymes function to suppress bladder tumor growth [20]. Interestingly, knockout of Hpa2 in xenopus was associated with increased Erk phosphorylation [21], possibly involving LOX reduction and providing a connection between Hpa2 and the Erk pathway. We found that LOX staining was associated inversely with tumor stage, approaching significance (p=0.08; Table 7), in agreement with earlier reports identifying LOX as a tumor suppressor that inhibits Ras [22, 23]. It should be noted, however, that according to more recent studies, LOX functions to promote tumor growth and metastasis, and is a target for the development of anti-cancer drugs [24]. Clearly, more work is required to resolve the role of LOX and LOX-related enzymes in bladder cancer.

**Table 7:** LOX staining intensity correlates inversely with tumor stage

Stage	LOX (%)		Total
	0+1	2+3	
I	2 (15)	11 (85)	13
II	10 (25)	29 (75)	39
III	7 (43)	9 (57)	16
Total	19	49	68

p=0.08.

LOX induction by Hpa2 may turn very important in pathological conditions other than cancer. Most relevant is urofacial syndrome (UFS), a rare autosomal recessive disease characterized by facial grimacing when attempting to smile and failure of the bladder to void completely, resulting in high risk for renal failure [25]. Importantly, biallelic mutations of *HPSE2* is held responsible for some cases of UFS in families from different ethnic groups [18, 26, 27]. Moreover, mice carrying a mutant Hpa2 exhibit bladder dysfunction [19, 27], associating with increased bladder fibrosis [19], and die within one month after birth [19]. Of note, mice deficient for LOXL1 had lower urinary tract dysfunction [28]. Thus, deficiency of Hpa2 in UFS patients may lead to decreased LOX enzymes, resulting in bladder dysfunction. In addition, LOX activity has been shown to be crucial for the development of elastic vessels [29]. For example, rat pups weaned from their mothers and fed the general LOX/LOXL inhibitor, β-aminopropionitrile (β-APN), form aneurysms [29]. In addition, LOX knockout mice die soon after birth [30], due to impaired vascular development that leads to an aneurysmal dilatation of the aorta and subsequent rupture [29, 30]. The relevance of LOX and vascular abnormalities to the lethal phenotype of Hpa2 deficient mice is yet to be determined.

Taken together, our results support the notion that Hpa2 function as a tumor suppressor in bladder cancer. This is the exact opposite of heparanase expression pattern, which is not detected in the normal bladder but is highly expressed in bladder carcinoma, correlating with disease progression [31, 32]. Thus, these two homolog proteins yield very different disease outcome in bladder cancer. We further show that Hpa2 function is not restricted to modulation of heparanase activity but apparently is involved in regulation of selected genes exemplified by LOX and E-cadherin.
